# Distinct stages of the intestinal bacterial community of *Ampullaceana balthica* after salinization

**DOI:** 10.3389/fmicb.2022.767334

**Published:** 2022-08-30

**Authors:** Carmen Kivistik, Kairi Käiro, Helen Tammert, Inna M. Sokolova, Veljo Kisand, Daniel P. R. Herlemann

**Affiliations:** ^1^Estonian University of Life Sciences, Center for Limnology, Tartu, Estonia; ^2^Department of Marine Biology, Institute for Biological Sciences, University of Rostock, Rostock, Germany; ^3^Department of Maritime Systems, Interdisciplinary Faculty, University of Rostock, Rostock, Germany; ^4^Institute of Technology, University of Tartu, Tartu, Estonia

**Keywords:** salinity, aquatic snail, Baltic Sea, *Ampullaceana balthica*, osmolarity, gastrointestinal bacteria

## Abstract

Environmental disturbances influence bacterial community structure and functioning. To investigate the effect of environmental disturbance caused by changes in salinity on host-protected bacterial communities, we analyzed the microbiome within the gastrointestinal tract of *Ampullaceana balthica* in different salinities. *A. balthica* is a benthic gastropod found in fresh- and mesohaline waters. Whereas the total energy reserves of *A. balthica* were unaffected by an increase of salinity to 3, a high mortality rate was detected after a shift from freshwater to salinity 6 suggesting a major disruption of energy homeostasis. The shift to salinity 6 also caused a change in the gastrointestinal bacterial community composition. At salinity 3, the bacterial community composition of different host individuals was related either to the freshwater or salinity 6 gastrointestinal bacterial community, indicating an ambivalent nature of salinity 3. Since salinity 3 represents the range where aquatic gastropods are able to regulate their osmolarity, this may be an important tipping point during salinization. The change in the intestinal microbiome was uncoupled from the change in the water bacterial community and unrelated to the food source microbiome. Our study shows that environmental disturbance caused by salinity acts also on the host-protected microbiome. In light of the sea-level rise, our findings indicate that salinization of the near-shore freshwater bodies will cause changes in organisms’ intestinal microbiomes if a critical salinity threshold (presumably ∼3) is exceeded.

## Introduction

Disturbances caused by changes in resources or in the physical environment lead to changes in species richness, community structure (Mackey and Currie, 2001; Svensson et al., 2009), and ecosystem functioning (Naeem et al., 1994; Hooper et al., 2005). Communities respond differently to disturbances depending on the disturbance type, length, intensity, and frequency, as well as the species tolerance capacity (Sousa, 1984; Eckert et al., 2019; Miller et al., 2021). The intestinal tract has long been recognized as an important site for host-microbe interactions. In healthy animals, gut microbial communities benefit host development, growth, homeostasis (Strasdine and Whitaker, 1963; Levy et al., 2017; Sommer et al., 2017), and nutrition by detoxifying secondary compounds in the food (Bhat et al., 1998; Dillon and Dillon, 2004). Animals, which may experience periodic compositional changes in diet or environment, have variable microbiome structures (Hooks and O’malley, 2017). Microbiome community composition can shift when disturbances are stronger than forces driving stability (Levy et al., 2017). A healthy microbiome could be replaced by those associated with dysbiosis (Hamdi et al., 2011; Levy et al., 2017). When the new microbiome community is not anymore able to support resistance to disturbances (Lozupone et al., 2012; Clark et al., 2015; Sommer et al., 2017; Ma, 2020), it can lead to changes in host health and fitness (Marasco et al., 2022). Since bacteria in the gastrointestinal microbiome are key players in host-associated microbiomes, it is crucial to understand the effect of disturbances and how bacterial communities respond to them (Allison and Martiny, 2008; Shade et al., 2011).

Salinity is an important physiological stress factor and a key variable explaining the global distribution patterns of bacteria, causing shifts in composition and affecting the functional performance of bacterial communities (Del Giorgio and Bouvier, 2002; Langenheder et al., 2003; Lozupone and Knight, 2007; Herlemann et al., 2011). Differential distribution of bacterial taxa along a salinity gradient implies that certain taxa are vulnerable to altered salinity so pulse disturbance in salinity favors habitat generalists with ecological adaptability and broad salinity tolerance (Székely and Langenheder, 2014). Salinity has been frequently used to investigate a disturbance in planktonic bacterial communities (e.g., Székely and Langenheder, 2014; Berga et al., 2017; Herlemann et al., 2017) since salinity fluctuations cause severe stress for organisms (Shetty et al., 2019) by shifting the cellular and tissue osmotic balance and negatively impacting key cellular processes (Prosser, 1991; Berger and Kharazova, 1997). Animals react to changes in salinity either by actively regulating the osmotic pressure of extracellular fluids around the physiologically optimal set-point (osmoregulator) or by adjusting the intracellular osmolarity to match the external osmolarity (osmoconformers) (Truchot, 1993). Osmoregulators have high energy demand for water and solute transport (Sokolova et al., 2012). In osmoconformers, the extra- and intracellular environment is maintained isosmotic to the external environment to prevent cell volume changes. This strategy is less energy-demanding but the organisms must cope with the shifts in osmotic homeostasis (Sokolova et al., 2012). Typically, euryhaline fresh- and brackish-water gastropods can osmoregulate at low salinities (< 100 mOsm corresponding to salinity < 3) and become osmoconformers at higher salinities (Jordan and Deaton, 1999).

Several studies have investigated the effects of disturbances on bacterial community composition and functioning (e.g., Shade et al., 2012; Berga et al., 2017); however, little is known about disturbance effects on host-associated bacterial communities. A study of salinity manipulation on osmoregulating fish showed that on each salinity level a unique set of dominating bacteria exists that rarely overlap along salinity gradients (Schmidt et al., 2015). Schmidt et al. (2015) noted that the changes in host-protected microbiomes were not correlated with corresponding changes in surrounding water bacterial communities, which suggests host-specific effects shaping the intestinal microbiome. In this study, we used a common pond snail *Ampullaceana balthica* (Linnaeus, 1758) to investigate the impact of disturbances on bacterial communities in a host-protected environment. *A. balthica* is a Palaearctic species widely distributed in Eurasia (Mandahl-Barth, 1938; Økland, 1990;Kerney, 1999) and North Africa (Van Damme, 1984; Glöer and Diercking, 2010). The snail prefers low-altitude freshwater bodies such as lakes, ponds, drainage ditches, and lentic zones of rivers, rich in nutrients and submerged vegetation (Glöer and Diercking, 2010). *A. balthica* feeds on detritus, periphyton, diatoms, and filamentous algae (Gordon et al., 2018) and can selectively forage for high-quality food particles (Fink and Von Elert, 2006). It is typically found in freshwater but can tolerate salinity up to 15 (Zettler et al., 2006).

Current climate change and anthropogenic activities cause coastal freshwater areas and inland freshwater bodies to become more saline, affecting the organisms living in these areas (Horton et al., 2014; Jeppesen et al., 2020). Therefore, the effect of environmental disturbance and especially salinity on bacterial communities in host-protected systems requires better understanding. Salinization can occur suddenly as a short pulse by extreme weather events or slow by sea level rise. In this study, we investigated the effect of disturbance (salinity and antibiotics) on the host gastrointestinal microbiome of *A. balthica* during a sudden change in experimental conditions and *in situ* representing conditions that have adapted over long periods. By comparing a sudden manipulation of the gastrointestinal microbiome with holobionts that have had a long exposure to increased salinity *in situ* we also improve the understanding of the adaptation mechanism for salinity. We hypothesized that (I) disturbance (increase in salinity/antibiotics) influences the bacterial community composition in the host environment and in the surrounding water; and (II) a long-term adaptation is necessary for the intestinal microbiome to cope with higher salinities. If salinity significantly influences the gastrointestinal microbiome, this would imply that the control of the host on its microbiome is weaker than the impact of salinity. If the response of the bacterial communities to the pulse manipulation has a negative effect compared to those observed in the holobionts adapted to different environmental salinity, we predict that a long adaptation to changes in salinity is necessary. Our alternative hypothesis was that salinity/antibiotics have no direct impact on the gastrointestinal microbiome. In this case, the host control over the microbiome is stronger than the effects of external disturbance.

## Materials and methods

### Sample collection and experimental setup

#### *In vivo* experiment

**FIGURE 1:**
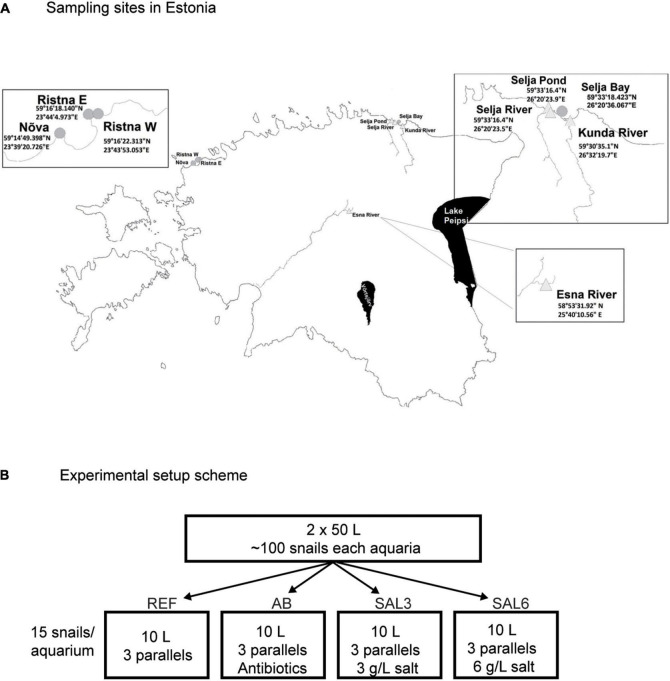
**(A)** Sampling sites in Estonia. Experiment snails were collected from the Esna River (ER). Coastal freshwater (*in situ* FW) sampling sites were: Selja pond (SP), Selja River (SR), and Kunda River (KR). Coastal brackish water sampling sites were: *in situ* SAL3 site was Selja Bay (SB), *in situ* SAL6 sites were Ristna western site (RW), Ristna eastern site (RE), and Nõva (NÕ). **(B)** Experimental setup scheme: REF, reference aquaria; AB, antibiotic manipulation aquaria; SAL3, salinity 3 manipulation aquaria; SAL6, salinity 6 manipulation aquaria.

*A. balthica* snails (∼200 specimens) were collected from the freshwater Esna River (ER; Figure 1A) in Estonia on 3 September, 2018. After the collection, the snails were kept in two 50 L acclimation aquaria with freshly collected 85 μm pre-filtered lake (Võrtsjärv) water under constant air supply at a controlled temperature of 16–17°C for 24 h. After 24 h, snails were divided (15 snails to each aquarium) to reference (REF), antibiotic amended (AB), salinity 3 (SAL3), and salinity 6 (SAL6) aquaria; all aquarium groups had three parallels (Figure 1B). The reference (REF) aquaria contained freshly filtered (85 μm) lake water. In the salinity 3 (SAL3) and salinity 6 (SAL6) aquaria, the salinity of the water was increased using commercially available Reef Salt (AQUA MEDIC). In the antibiotics aquarium (AB), which was a disturbance control testing if the intestinal bacterial community responds to manipulation, freshwater was amended with ampicillin (5 mg/L) and streptomycin (5 mg/L). All aquaria contained a 2-cm layer of sandy sediment from Lake Võrtsjärv (sieved through a 0.5-mm mesh size plastic sieve) and stones and pebbles with natural biofilm as a food source. Aquaria were constantly supplied with air and held at 16.1–17.5°C for 8 days. Water conditions were monitored daily using a YSI ProDSS multisensor (Supplementary Figure 1).

At the beginning of the experiment, before any manipulation (day 0), water from each aquarium was sampled by filtering 3 × 100 mL through a 0.2-μm Durapore membrane filter (Millipore) and 14 *A. balthica* snails (day 0 snails - 8 for 16S rRNA gene analysis and 6 for energy reserve measurements) were shock frozen. In addition to the day 0 samples, water samples (3 × 100 mL from each aquarium) were taken on day 1 and day 8 of the experiment from each aquarium (*n* = 26). Snails from the aquaria were collected on day 8 for 16S rRNA gene analysis (*n* = 26) and energy reserve estimation measurements (*n* = 34). Snails for 16S rRNA gene analysis were starved for 24 h in sterile falcon tubes under conditions identical to the experiment aquaria to minimize the occurrence of transient bacteria (Van Horn et al., 2012). The feces were collected afterward from the tubes and stored at −80°C for further analysis.

#### *In situ* samples

*A. balthica* snails from Estonian coastal area sites with fresh- and brackish (salinity 3 and salinity 6) water conditions were collected on 17–18 June 2019 (Figure 1A). The *in situ* freshwater sampling sites were Selja pond (SP), Selja River (SR), and Kunda River (KR). The sampling site with salinity 3 included Selja Bay (SB) and sampling sites with salinity 6 were Nõva (NÕ), Ristna western site (RW), and Ristna eastern site (RE).

Snails collected from the freshwater and coastal brackish areas were starved for 24 h in their native water pre-filtered through 0.22 μm pore size Sterivex™ filters, to reduce the number of food-derived microbes in the transient microbiome. The Sterivex™ filters were used for water bacterial community analysis. Collected snails were divided for 16S rRNA gene analysis (*n* = 18) and energy reserve measurements (*n* = 21), shock frozen in liquid nitrogen and stored at −80°C for further analysis. Biofilm samples were collected from all studied sites by scraping the stones or pebbles, immediately shock frozen in liquid nitrogen, and stored at −80°C.

### Lipid, carbohydrate, and protein measurements

To estimate the energy reserves, we measured lipid, carbohydrate, and protein content in snail tissues as described elsewhere (Haider et al., 2018). In brief, the frozen snails were thawed for 10 min at room temperature and removed from the shell with tweezers. The snails were frozen again in liquid nitrogen and powdered using a sterilized mortar. The tissue powder was placed in 2 mL microcentrifuge tubes. For lipid content analysis, approximately 30 mg of tissue powder was added to 3 mL of chloroform: methanol mixture (1:2, v:v) and incubated for 5 min with periodic vigorous mixing. The mixture was centrifuged at 3000 × *g* for 5 min at room temperature and the supernatants dried out at 100°C. Sunflower oil in acetone was used as a standard. The dry samples were solubilized with concentrated sulfuric acid (H_2_SO_4_) and mixed with a vanillin reagent. The absorbance of samples and standards was measured at 490 nm using a FLUOstar^®^ Omega microplate reader. For the determination of the carbohydrate and protein content, ∼50 mg of tissue powder was mixed in 0.5 mL of distilled water with 0.1% Triton (1:10 tissue mass to volume). Cells were lysed by three rapid freeze-thaw cycles of 5 min at −80°C followed by 5 min in a 37°C water bath and centrifuged at 3000 × *g* for 3 min at room temperature. The supernatant was used for carbohydrate and protein measurements. Carbohydrate concentrations were measured using the phenol-sulfuric acid method with glucose as a standard (Masuko et al., 2005). To calculate the carbohydrate content (in glucose equivalents), absorbance was measured at 492 nm using a FLUOstar^®^ Omega microplate reader. The soluble protein content was determined using the Bradford assay with bovine serum albumin (BSA) as a standard by measuring absorbance at 595 nm using the FLUOstar^®^ Omega microplate reader.

Total energy reserve was calculated by transforming the measured protein, lipid, and carbohydrate content into energy equivalents using their respective energy of combustion: 24 kJ g^–1^ for proteins, 39.5 kJ g^–1^ for lipids, and 17.5 kJ g^–1^ for carbohydrates (Gnaiger, 1983).

### 16S rRNA gene analysis

#### Snail preparation for 16S rRNA gene analysis

For the 16S rRNA gene analysis, the frozen snails were allowed to thaw for 10 min at room temperature and cleaned with 90% ethanol. The soft body of the snail was removed from the shell with tweezers on a sterile Petri dish without breaking the shell, avoiding contamination from bacteria living on the shell. The gastrointestinal tract of the snail was dissected, placed into the 2 mL Eppendorf tube and frozen at −80°C for further analysis.

#### DNA extraction process

DNA of the snails’ gastrointestinal microbiome, biofilm, feces, and water filters was extracted using the phenol:chloroform method according to the modified protocols from Lueders et al. (2004) and (Weinbauer et al., 2002) as described in detail in Kivistik et al. (2020). In brief, to release the DNA, we used mechanical bead beating and thermal disruption (65°C for 1 h). For the precipitation, we used phenol:chloroform:isoamyl alcohol (25:24:1) at pH 8 and chloroform:isoamyl (24:1) to separate the DNA from the cell debris. The RNA was removed after chloroform/phenol extraction using RNase A (Qiagen). The subsequent purification steps included ice-cold isopropanol and 96% ethanol. The remaining pellet was resuspended in the 50 μL AE buffer (10 mM Tris-Cl, 0.5 mM EDTA; pH 9.0) (Qiagen). The amount and quality of the DNA were estimated using a NanoDrop™ UV-Vis spectrophotometer. The DNA sequences were amplified using the primers Bakt_341F and Bakt_805R according to a modified protocol of Herlemann et al. (2011) using 30 PCR cycles and processed as described in Kivistik et al. (2020).

#### Sequencing

Amplicons were purified using PCR Kleen (Bio-Rad). Illumina TrueSeq adapters and P5/P7primers tags were added to amplicons in the second PCR reaction and sequenced at FIMM, University of Helsinki, Finland. A total of 7,353,519 reads were generated for 66 samples by Illumina MiSeq sequencing using PE250 chemistry (MiSeq Reagent Kit v2). The resulting sequences were processed using Trimmomatic (V0.36) (Bolger et al., 2014) to remove Illumina-specific sequences and regions with low sequence quality (average quality score < Q20). PCR primers were removed using the default values in Cutadapt (V2.3) (Martin, 2011). The reads were paired (16 bp overlap, minimum length 300 bp) using the VSEARCH tool (Rognes et al., 2016). These were then taxonomically assigned using the SILVA next-generation sequencing (NGS) pipeline (Glöckner et al., 2017) using the SILVAngs analysis platform release version 138 (Pruesse et al., 2007). SILVAngs analysis platform performs additional quality checks according to SINA-based alignments (Pruesse et al., 2012) with a curated seed database in which PCR artifacts or non-SSU reads are excluded. The longest read serves as a reference for taxonomic classification using a BLAST (version 2.2.30 +) search against the SILVA SSURef dataset. The classification of the reference sequence of each cluster (98% sequence identity) is mapped to all members of the respective cluster and to their replicates. Non-bacterial sequences such as chloroplasts, mitochondria, eukaryotes, and Archaea were excluded because the primer set employed in the analysis has only limited coverage. The raw reads of the 16S rRNA genes were deposited at the NCBI SRA under bioproject PRJNA724976, accession number SAMN18865776-SAMN18865895.

### Host species taxonomic identification

To verify the species’ taxonomic identification, an internal transcribed spacer 2 (ITS2) analysis of *A. balthica* was conducted. The DNA was amplified as described in (Kivistik et al., 2020) using the primers LT1 (Bargues et al., 2001) and ITS2-Rixo (Almeyda-Artigas et al., 2000). The PCR protocol was 94°C for 4 min for denaturation followed by 35 cycles of 94°C for 30 s, 56°C for 30 s, 72°C for 1 min, and the final extension at 72°C for 7 min. The amplicon was purified using PCR Kleen (Bio-Rad) and Sanger sequenced by the sequencing facility at Tartu University, Estonia. The sequences from the ITS2 region were quality-checked using the software Chromas (Technelysium Pty Ltd., Australia); forward and reverse reads were assembled after low-quality reads were discarded. All the sequences were imported into ARB (Ludwig et al., 2004) to calculate a maximum likelihood phylogenetic tree (PhyML). Bootstraps were calculated using the RAxML 7.0.3 (Stamatakis, 2006) rapid bootstrap analysis with 1000 runs as implemented in ARB and added to the PhyML tree. The ITS reads were deposited at EBI under accession number OA985092-OA985113.

### Statistical analysis

The number of reads per sample varied between 1,004 and 86,933 reads, therefore, the data were normalized by cumulative sum scaling (CSS) using the R (Ihaka and Gentleman, 1996) package metagenomeSeq (Paulson et al., 2013). Bacterial α-diversity is represented by the Chao1 index calculated with R using the “phyloseq” package (Mcmurdie and Holmes, 2013). For testing the sample groups’ homoscedasticity, we used Levene’s test from calculated energy content and Chao1 means. A one-way ANOVA test with an additional *post hoc* Tukey’s Honest Significant Differences (HSD) test was used to calculate significant differences between the number of OTUs in the samples and differences in total energy reserves. For the coastal snail samples’ total energy reserve, we used Welch’s ANOVA test with an additional Games-Howell *post hoc* test. The mean difference in community dissimilarity among the different treatments was determined by beta-dispersion analysis in PAST software, package version 4.07 (Hammer et al., 2001). Variations in bacterial community structure were characterized in a principal coordinate analysis (PCoA) using the Bray–Curtis dissimilarity in the “vegan” community ecology package of R (Oksanen et al., 2013) and PAST software package version 4.07 (Hammer et al., 2001) for visualization. The one-way permutational multivariate analysis of variance (PERMANOVA test) with Bray-Curtis dissimilarity was used to calculate differences between bacterial community compositions among analyzed sample groups. A linear discriminant analysis effect size (LEfSe) tool (Segata et al., 2011) was used to identify bacterial groups with multi-class analysis; the “One against all” was used with default settings (α = 0.05, N permutations = 1,000). OTUs identified in the LEfSe as significantly enriched were defined as indicator OTUs.

## Results

### Host body energy content

**FIGURE 2:**
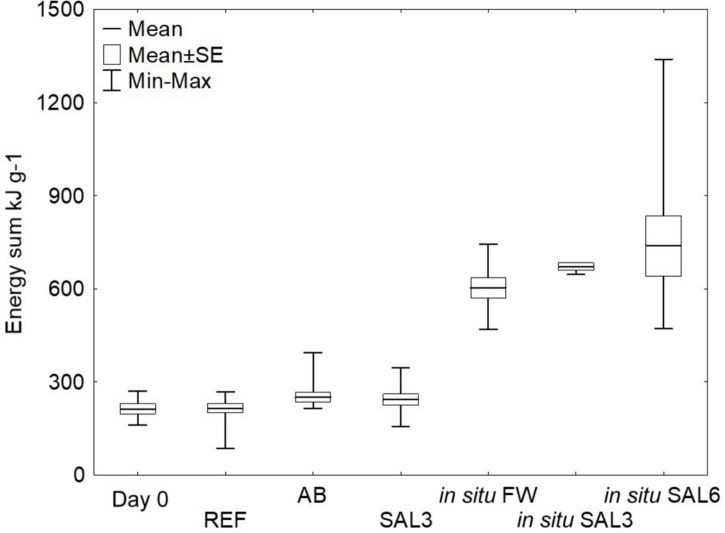
Total energy pool (kJ g^–1^) of the snails from the experiment and *in situ* sampling sites. Total energy pool kJ g^– 1^ calculated from the lipid, carbohydrate, and protein content of the snail tissues. Day 0: snails from freshwater Esna River on experiment’s day 0 (*n* = 6); REF: reference snails from non-manipulated freshwater aquaria (*n* = 11); AB: snails from antibiotics manipulated aquaria (*n* = 11); SAL3: snails from aquaria with raised salinity to 3 (*n* = 12). *In situ* FW: snails collected from natural freshwater sites (SP, SR, KR; *n* = 9); *in situ* SAL3: snails collected from a natural site with salinity 3 (SB, *n* = 3); *in situ* SAL6: snails collected from natural sites with salinity 6 (RE, RW, NÕ; *n* = 9).

The snail mortality rate in salinity 6 aquaria was 95%; therefore, surviving snails from salinity 6 (*n* = 3) were excluded from the energy calculations and the rapid salinity rise from freshwater to salinity 6 was considered highly stressful for the snails. For testing the experimental sample groups’ homoscedasticity, we used Levene’s test from calculated energy content means, which was not significantly different (*p* > 0.05). Tukey’s HSD test showed that the freshly collected (day 0) ER *A. balthica* samples had a lower lipid energy content (0.7 kJ g^–1^ SE ± 0.3, *p* < 0.01) compared to samples from REF (6.9 kJ g^–1^ SE ± 0.2), AB (6.6 kJ g^–1^ SE ± 0.2) and SAL3 (5.5 kJ g^–1^ SE ± 0.2) aquaria (Supplementary Table 1). The lipid concentration decreased in samples from manipulated SAL3 *A. balthica* compared to the reference samples. The protein-associated energy content was lower on day 0 *A. balthica* (162.6 kJ g^–1^ SE ± 1.3, *p* < 0.01, Supplementary Table 1) compared to REF (178.4 kJ g^–1^ SE ± 1), AB (178.4 kJ g^–1^ SE ± 0.9) and SAL3 snails (184 kJ g^–1^ SE ± 0.9). The calculated energy reserve stored in carbohydrate compounds did not show any significant difference between the studied groups of *A. balthica*. The average total energy reserve (calculated as the energy content of lipids, proteins, and carbohydrates per gram of tissue) was 213 kJ g^–1^ for day 0 *A. balthica*, 215 kJ g^–1^ for reference, 250 kJ g^–1^ for AB treated, and 242 kJ g^–1^ for salinity 3 treated samples (Figure 2). Tukey’s HSD test did not show any significant difference in the total energy reserves between the studied groups of *A. balthica*.

The total energy reserves of the snail collected from the field sites with different salinities (SP, SR, KR, SB, NÕ, RW, and RE) were higher compared to ER snails on day 0 or those exposed to different experimental treatments in the laboratory (*p* < 0.01). The average energy content was 604 kJ g^–1^ for *in situ* freshwater snails, 671 kJ g^–1^ for *in situ* salinity 3 samples, and 738 kJ g^–1^ for *in situ* salinity 6 samples (Figure 2). Welch’s ANOVA test with an additional Games-Howell *post hoc* test did not show any significant difference for the total energy content between the field-collected snails from the freshwater, salinity 3 and salinity 6 sites.

### Host species taxonomic identification

**FIGURE 3:**
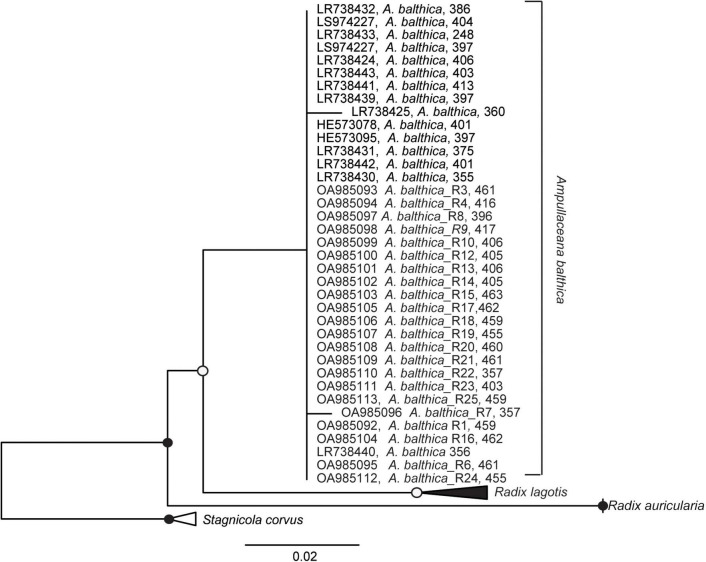
Maximum likelihood tree of nuclear marker internal transcribed spacer 2 (ITS2) sequence based on 398 sequence columns with ITS2 sequences using *Stagnicola corvus* as an outgroup. Sequences OA985092-OA985113 were derived in this study from *Ampullaceana balthica* sampled in the Esna River and the coastal sampling sites of Estonia. Filled dots represent a bootstrap value of 100% and empty dots of a bootstrap > 75%.

Since the total energy pools between *in situ* snails and experimental snails differed significantly, and snails from natural sites were tolerating higher salinity with no extra energy demand, we analyzed the internal transcribed spacer 2 (ITS2) biomarker regions to test whether this physiological variation might be due to the presence of cryptic species. Our analysis confirmed that the snails used in the experiments and from coastal regions (SP, SR, KR, SB, NÕ, RW, and RW) belong to *A. balthica* with almost identical ITS sequences (99% identity Figure 3).

### Host gastrointestinal bacterial richness

**FIGURE 4:**
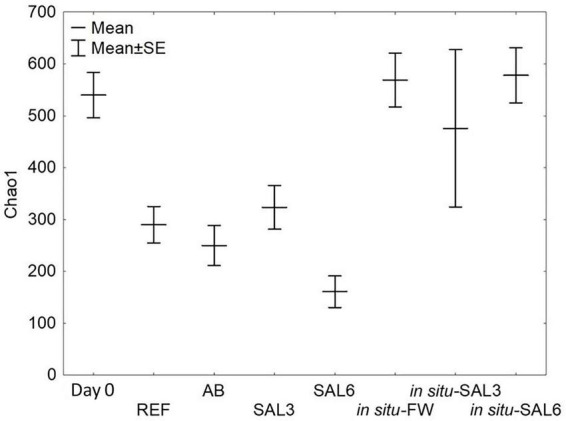
Bacterial α-diversity represented by the Chao1 index. Day 0: snails from freshwater Esna River on experiment’s day 0; REF: reference snails from non-manipulated freshwater aquaria; AB: snails from antibiotics manipulated aquaria; SAL3: snails from aquaria with raised salinity to 3, SAL6: snails from aquaria with raised salinity to 6. *In situ* FW: snails collected from natural freshwater sites; *in situ* SAL3: snails collected from a natural site with salinity 3; *in situ* SAL6: snails collected from natural sites with salinity 6.

Among laboratory experiments and *in situ* coastal site samples, SILVA NGS classified 3,435 operational taxonomic units (OTUs) from a total of 3,195,039 sequences in 75 different phyla. For testing the sample groups’ homoscedasticity, we used Levene’s test from bacterial Chao1 index means which was not significantly different (*p* > 0.05). Transferring snails collected from ER to the reference aquarium (REF) resulted in a decrease in the bacterial Chao1 mean (ER: 540 SE ± 48.3; REF: 290 SE ± 48.3, *p* < 0.01, Figure 4). Further treatment with antibiotics or elevated salinity in the laboratory caused no significant change in the Chao1 mean of the *A. balthica* intestinal microbiome (AB: 250 SE ± 51.6, SAL3: 323 SE ± 51.6 and SAL6: 161 SE ± 78.8; *p* > 0.01). Similar to the manipulation experiments, the bacterial Chao1 means of the field-collected freshwater snails (SP, SR, and KR: Chao1 = 569 SE ± 51.6), salinity 3 snails (SB: 476 SE ± 96.6), and salinity 6 snails (NÕ, RE, RW: 578 SE ± 39.4) (Figure 4) were not significantly different (Tukey’s HSD test *p* > 0.01).

Interestingly, the detected bacterial diversity of the experiment’s day 0 snails (Chao1 mean 540 SE ± 48.3) was comparable to the bacterial diversity of the snails collected from the freshwater coastal area *in situ* (569 SE ± 51.6, *p* > 0.01, Figure 4). Also, Chao1 mean of *in situ* SAL3 (578 SE ± 39.4) and experimental SAL3 aquaria snails bacterial diversity (323 SE ± 51.6) were not significantly different (*p* > 0.05). An exception was the Chao1 mean of *in situ* SAL6 snails bacterial diversity, which was higher (578 SE ± 39.4, *p* < 0.01) compared to laboratory experiment SAL6 aquaria snails bacterial diversity (161 SE ± 78.8).

### Host gastrointestinal bacterial community composition

**FIGURE 5:**
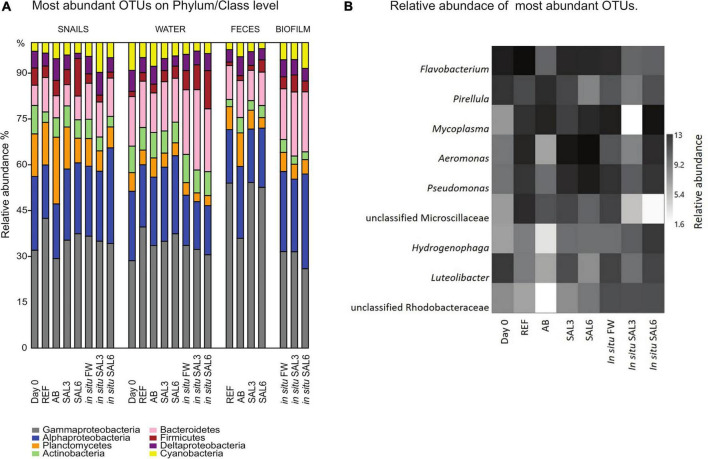
**(A)** Relative abundance (%) of most abundant operational taxonomic units (OTUs) on Phylum/Class level in all analyzed samples. **(B)** Relative abundance of most abundant OTUs of laboratory experiment snail samples and *in situ* coastal area snail samples. Day 0: snails from freshwater Esna River on experiment’s day 0; REF, reference snails from non-manipulated freshwater aquaria; AB, snails from antibiotics manipulated aquaria; SAL3, snails from aquaria with raised salinity to 3, SAL6, snails from aquaria with raised salinity to 6. *In situ* FW, snails collected from natural freshwater sites; *in situ* SAL3, snails collected from natural site with salinity 3; *in situ* SAL6, snails collected from natural sites with salinity 6.

The bacterial community composition on phylum/class level was comparable among all analyzed samples (Figure 5A). Samples were dominated by *Proteobacteria* (34–71%) of which *Gammaproteobacteria* (69–73%) and *Alphaproteobacteria* (9–40%) were the dominant classes. Furthermore, *Planctomycetes* (2.4–28%), *Bacteroidetes* (3.4–15.8%), *Firmicutes* (1–15%), *Actinobacteria* (1–10%), and *Cyanobacteria* (1-9%) were found in high abundance.

At the genus level, the most abundant bacteria in all analyzed snail gastrointestinal samples were *Flavobacterium, Pirellula, Mycoplasma, Aeromonas, Pseudomonas*, uncultured Microscillaceae OTU, *Hydrogenophaga, Luteolibacter*, and unclassified Rhodobacteraceae OTU (Figure 5B). The most abundant OTUs in experiment’s day 0 snail samples were *Flavobacterium* (0.5–1.0%), unclassified Rhizobiales (0.5–0.9%), and *Pirellula* (0.5–0.9%). After transferring snails to REF aquaria, the bacterial community was still dominated by *Flavobacterium* (1.0–2.9%), but *Acinetobacter* (1.0–2.3%), and *Aeromonas* (0.8–5.0%) became more abundant. The snails’ microbiome from the SAL3 aquaria was dominated by similar bacteria (*Flavobacterium* 0.7–3.2%, *Acinetobacter* 0.7–3.6%, and *Aeromonas* 0.7–3.5%) as the REF aquaria snails’ microbiome. Snails’ microbiomes from SAL6 aquaria were dominated by *Aeromonas* (1.7–2.4%), and *Shewanella* (1.7–2.1%) along with *Pseudomonas* (1.4–2.0%). The antibiotic manipulation caused *Mycoplasma* (0.7–1.9%), *Pirellula* (0.8–1.8%), and *Chryseobacterium* (0.6–1.8%) to become more abundant. Genera that dominated the *in situ* freshwater snail samples (SP, SR, and KR) were *Mycoplasma* (0.3–1.0%), unclassified Microscillaceae (0.3–0.9%), and *Flavobacterium* (0.5–0.8%). The relative abundance of the dominant OTUs in *in situ* salinity 3 (SB) snail samples was *Rhodobacter* (0.5–0.9%), unclassified Rhodobacteraceae (3.8-9.9%), and *Hydrogenophaga* (0.4–0.8%). The relative abundance of the dominant OTUs in *in situ* salinity 6 (NÕ, RW, and RE) snail microbiome was *Mycoplasma* (0.5–2.2%), unclassified Rhizobiaceae OTU (0.4–1.7%), and *Pseudomonas* (0.5–1.3%). In addition to the abundant bacterial genera, we identified characteristic OTUs for snail sample groups using LEfSe (Table 1).

**TABLE 1:** Characteristic operational taxonomic units (OTUs) for snail sample groups using linear discriminant analysis effect size (LEfSe) tool.

Day 0 snail	REF	AB	SAL3	SAL6	*In situ* freshwater	*In situ* SAL3	*In situ* SAL6
*Tabrizicola*	*Acinetobacter*	*Chryseobacterium*	*Acinetobacter*	*Shewanella*	*Neochlamydia*	Candidatus Competibacter	*Spiroplasma*
*Rhodobacter*	*Aeromonas*	*Ensifer*	*Aeromonas*	*Aeromonas*	Candidatus Competibacter	Phormidium ETS-05	
	*Mycoplasma*		*Tabrizicola*	*Mycoplasma*			
			*Shewanella*				

**FIGURE 6:**
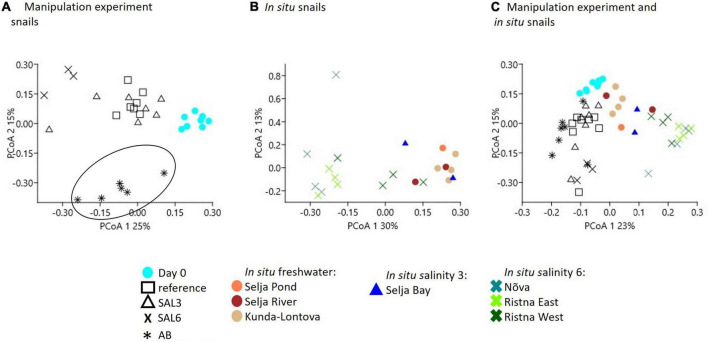
Principal coordinate analysis based on the Bray-Curtis dissimilarity of bacterial community composition on operational taxonomic unit (OTU) level. **(A)** Experiment snail samples (AB samples are surrounded with the circle to illustrate the clustering from the other samples), **(B)** *in situ* coastal area snail samples, **(C)** experiment, and *in situ* coastal area snail samples.

A multivariate dispersion analysis to determine the variability in species composition did not show any significant differences within the experimental snail samples nor within snails collected from *in situ* conditions (*p* = 0.194, *p* = 0.020, respectively, Supplementary Figure 2). To visualize the differences in the host-associated bacterial community composition between the experiment and snails collected from *in situ* conditions, we employed PCoA (Figure 6). The PCo analysis indicated the difference in the microbiome of the day 0 snails relative to the snails maintained in experimental aquaria (Figure 6A). PERMANOVA test confirmed the difference between microbiomes of experiment’s day 0 snails and snails from aquaria (*R*^2^ = 85%, *p* < 0.01). The PCo2 separated antibiotic-treated snail microbiomes from the rest (*R*^2^ = 86%, *p* < 0.01; Figure 6A). Furthermore, the gastrointestinal bacterial community of salinity 6 treated snails was significantly different (*R*^2^ = 89%, *p* < 0.01) from the rest of the experiment snails (Figure 6A). Interestingly, five SAL3 aquaria snails’ microbiomes (Figure 6A) grouped with the snails from REF (Figure 6A) and two – with SAL6 samples (Figure 6A). Similar to the experiment, the *in situ* SAL6 samples differed significantly from the *in situ* freshwater (SP, SR, and KR) and *in situ* SAL3 (SB) snails microbiomes (*R*^2^ = 76%, *p* < 0.01, Figure 6B). The difference between *in situ* freshwater and *in situ* SAL3 snails’ microbiome was minor (*p* > 0.01). Comparison between the *in situ* snail’s and laboratory experiment snail’s gastrointestinal bacterial communities revealed a separation on the PCo1 (PERMANOVA *R*^2^ = 79%, *p* < 0.01, Figure 6C). The experiment day 0 and *in situ* freshwater bacterial communities (Figure 6C) clustered together. However, the microbiome of *in situ* fresh- and brackish site snails differed from experiments REF, AB, SAL3, and SAL6 aquaria snails (*R*^2^ = 60%, *p* < 0.01).

### Bacterioplankton community composition

The aquarium water bacterial α-diversity, represented by Chao1 mean, was 906 (SE ± 45.7) on experiment day 1 and 705 (SE ± 52.1) on day 8 and did not show significant difference across all aquaria (REF, AB, SAL3, and SAL6). The Chao1 mean of day 0 water samples 865 (SE ± 33.1) did not differ from day 1 and day 8 water samples’ bacterial diversity. The Chao1 mean was 1,055 (SE ± 45.6) for *in situ* freshwater water samples, 993 (SE ± 69.6) for *in situ* SAL3 samples, and 1,143 (SE ± 69.6) for *in situ* SAL6 samples. According to Tukey’s HSD test, the bacterial richness of water samples was not significantly different between the coastal area sampling sites (Supplementary Figure 3).

The most abundant bacterioplankton species in aquaria water samples and in *in situ* water samples were *Flavobacterium* and *Limnohabitans*. However, the aquarium water was also enriched with LD29, *Methylocystis*, and *Cyanobium* PCC-6307, and water from the coastal sites was enriched with *Fluviicola, Sporichthyaceae* hgcI clade, and *Pseudomonas.* Surprisingly, even though the PCo1 separated the day 1 and day 8 water samples, the bacterial community composition was similar according to PERMANOVA test (REF: *R*^2^ = 54%, *p* = 0.09, *n* = 5; AB: *R*^2^ = 23%, *p* = 0.2, *n* = 4; SAL3: *R*^2^ = 43%, *p* = 0.1, *n* = 6), except for SAL6 aquaria day 1 and day 8 samples (*R*^2^ = 51%, *p* < 0.01, *n* = 8) (Supplementary Figure 4A). However, a larger sample size might have shown a clearer result. Water bacterial communities from the *in situ* fresh- and brackish sites differed from each other showing site-specific bacterial community compositions (Supplementary Figure 4B).

### Impact of water and food sources on the gastrointestinal bacterial community composition

**FIGURE 7:**
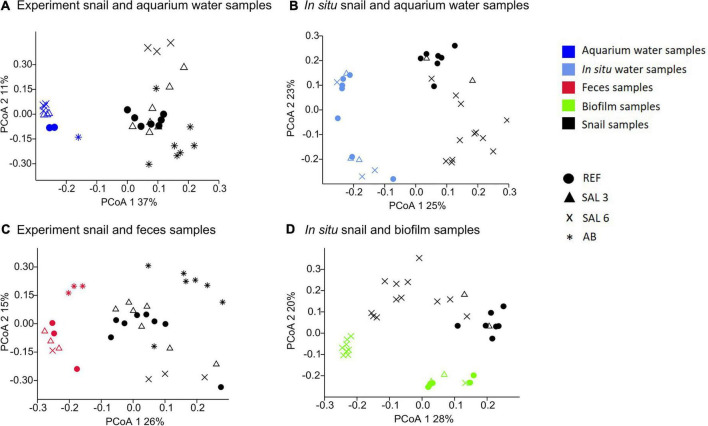
Principal coordinate analysis of bacterial community composition based on the Bray-Curtis dissimilarity on operational taxonomic unit (OTU) level. **(A)** Experiment snail and aquarium water samples, **(B)** *in situ* coastal area snail and water samples, **(C)** experiment snail and fecal samples, **(D)** *in situ* coastal area snail and biofilm samples.

The most abundantly found bacteria in biofilm samples differed from those in the water and the snail microbiome and included uncultured *Rhodobacteraceae*, *Flavobacterium*, uncultured *Saprospiraceae, Hydrogenophaga*, and *Porphyrobacter*. The most abundant bacteria found in the snail feces were *Pseudomonas*, *Acinetobacter*, *Flavobacterium, Simplicispira*, and *Acidovorax.* PCoA (Figures 7A,B) indicated a clear difference between gastrointestinal tract microbiome and water samples for both experiments and *in situ* samples with no similarity between water–microbiome pairings from the same aquarium or *in situ* sampling site. The PERMANOVA test with the Bray–Curtis dissimilarity confirmed that the bacterial communities of snail microbiome samples were different from water samples (*R*^2^ = 70%; *p* < 0.01; Figures 7A,B), biofilm (*R*^2^ = 54%; *p* < 0.01), and feces (*R*^2^ = 5%; *p* < 0.01) (Figures 7C,D).

## Discussion

Communities respond differently to disturbances depending on the disturbance type, length, intensity, and frequency, as well as the species tolerance capacity (Sousa, 1984). We investigated the impact of the disturbance caused by salinity upshift on the gastrointestinal microbiome of *A. balthica* to understand the capacity of the host to protect its microbiome. Although this investigation is based only on one species, it improves our understanding of host-microbiome interactions in a changing environment especially in view of further sea level rise, change in land use, and extreme weather events affecting coastal and inland freshwater communities.

### Impact of the salinity manipulation on the hosts’ total energy pool

In this study, we used lipid, carbohydrate, and protein compound measurements from the snail tissue to estimate the total energy pool of the specimens. The experimental shift from freshwater to salinity 6 caused high mortality (95%) after 8 days of saline stress. The high mortality indicated that the freshwater population of *A. balthica* used for the laboratory experiments has limited capacities to osmoregulate during a sudden salinity increase. However, *A. balthica* can be found at a salinity of >15 in coastal areas, demonstrating its general capability to adapt to elevated salinity (Zettler et al., 2006). The high levels of energy reserves of the snails taken at salinity 6 on the coastal sites indicated that the long-term adaptation to elevated salinity is not associated with the impaired energy status (Figure 2). However, lower energy availability (such as has been observed in the laboratory-maintained snails in our present study) might impair the ability of snails to survive an acute increase of salinity to 6. Although we could not assess the energy status of the snails during the laboratory experiments with salinity 6, high mortality in this experimental group suggests a major disruption of metabolism and energy homeostasis (Sokolova, 2021). The sudden shift from freshwater to salinity 3 showed no change in the total energy reserves. Differences in the response to salinity 6 and salinity 3 may be connected with the different osmoregulation strategies employed by the snails at these salinities (Jordan and Deaton, 1999). Many euryhaline gastropods osmoregulate in the lower part of their salinity tolerance range (typically < 100 mOsm corresponding to salinity < 3) and osmoconform at higher salinities (Jordan and Deaton, 1999). Thus, the transition to the osmoconforming strategy combined with the low levels of energy reserves in the laboratory-maintained *A. balthica* might have overstressed the organism’s capacity to maintain intracellular homeostasis at salinity 6.

The body energy content of the snails maintained for 8 days in the aquaria was higher than in those collected from the freshwater ER (day 0 snails, Figure 2). Furthermore, the energy reserves of the day 0 snail samples were lower than in the snails collected from *in situ* coastal sampling sites. This suggests a stressful situation in the freshwater ER during sampling. Data from the Estonian Weather Service (www.ilmateenistus.ee) indicate that the summer of 2018 was unusually warm and dry in Estonia. This could explain the lower total energy pool measurements of day 0 snails compared to the total energy pool of the samples collected from the Estonian coastline from 2019. *A. balthica* has a low tolerance to increasing water temperatures (Cordellier and Pfenninger, 2009) and their optimal temperature is 16-20°C with elevated mortality and reproductive failure above 24°C (Johansson and Laurila, 2017). Therefore, a temperature rise of >20°C for an unusually long period in rivers, observed in the summer of 2018, could cause high-stress levels and may be responsible for the impaired energy status of the experiment’s day 0 snails. In the laboratory experiments, eight days of recovery under optimal temperature conditions (∼17°C) restored the energy balance and increased the deposition of energy reserves in *A. balthica*.

### Impact of disturbance on the host gastrointestinal bacterial community

#### Impact of salinity on host gastrointestinal bacterial richness

Changes in salinity (freshwater or marine water becoming brackish) cause a reduction in invertebrate richness (Remane, 1934) and phytoplankton diversity (Olli et al., 2019). However, in certain cases, a peak of microbial species occurring in intermediate salinities has been described (Telesh et al., 2011; Pavloudi et al., 2017). Our results showed evidence for a similar Chao1 mean of the experiment’s day 0 snails and the field-collected snails from the coastal freshwater sites (SP, SR, and KR) (Figure 4). Furthermore, Chao1 means did not differ between the samples from different experimental conditions or between different *in situ* samples (Figure 4). This is consistent with the earlier findings showing that the pelagic and benthic bacterial richness is rather constant along environmental salinity gradients (Herlemann et al., 2011, 2016; Berga et al., 2017; Klier et al., 2018). Previous research in host-associated systems has shown that the freshwater-saltwater change did not affect the bacterial α-diversity in *Salmo salar L.* (Dehler et al., 2017) or *Theodoxus fluviatilis* (Kivistik et al., 2020) even with the salinity change from 0.5 to 28.

However, a reduction of Chao1 was observed by transferring day 0 snails to the REF aquarium for 8 days (Tukey’s HSD test, *p* < 0.01) and the Chao1 index of the aquarium snails was lower than the Chao1 mean of coastal area samples. This may indicate an experimental-driven response. A decline in bacterial richness and changes in the bacterial community composition due to an organism’s transfer from natural conditions to aquaria settings has been observed previously (Pratte et al., 2015; Kivistik et al., 2020). The changes in bacterial communities due to the transfer to closed systems have been ascribed to grazing (Jürgens and Güde, 1994), changes in the carbon quality (Herlemann et al., 2014), missing replacement of bacteria (Ionescu et al., 2015), alternative strategies to acquire carbon (Herlemann et al., 2019), and accumulation of toxic metabolites. In addition, the change in the conditions of the host due to the shift from a river environment to a pond-like environment in the aquarium could influence the bacterial richness. Overall, our results suggest that the number of bacteria observed in a host-protected environment is influenced by other factors than by the changes in salinity.

#### Impact of the salinity on host gastrointestinal bacterial community composition

The bacterial 16S rRNA gene profiling revealed a diverse community predominantly derived from *Gammaproteobacteria, Alphaproteobacteria, Bacteroidetes, Firmicutes, Actinobacteria, Planctomycetes*, and *Cyanobacteria*. The high abundance of *Gammaproteobacteria* and *Alphaproteobacteria* has been previously found in invertebrate gastrointestinal microbiomes including *Achatina fulica* (Pawar et al., 2012), Diplopoda, *Cylindroiulus fulviceps* (Knapp et al., 2009), and oysters (King et al., 2012). However, the analysis at a finer taxonomic resolution (OTUs) indicates that the composition of the gastrointestinal bacterial community of *A. balthica* changes in response to elevated salinity. A clear difference between freshwater and salinity 6 treated snails was found, but interestingly, the bacterial community composition of snails from salinity 3 seems to have the ability to develop in opposite ways to resemble either the freshwater or salinity 6 samples. This pattern was apparent in the snails from the experimental aquarium SAL3 as well as in those collected *in situ* at the salinity 3 (SAL3) site. In pelagic environments, gradual changes in the bacterial community composition at increasing salinities have been previously observed (Herlemann et al., 2011) similar to the changes found in the water samples in our present experiment. Our results suggest that in a host-protected environment of *A. balthica*, the gastrointestinal bacterial communities show distinct salinity-associated profiles, either freshwater-like or salinity 6-like. This may be due to the gastropods’ ability to maintain stable internal osmolarity below salinity 3 and transition to osmoconformity at higher salinities (Jordan and Deaton, 1999). The presence of both types of gastrointestinal bacterial communities in the snails acclimated or adapted to salinity 3 might be due to the individual variability of the hosts’ osmoregulatory capacities, so that some individuals maintain the internal osmolarity similar to that found in the freshwater, while others switch to osmoconformity. This hypothesis requires further investigation. Overall, our bacterial community composition analysis partially contradicts the first hypothesis of our present study that snails collected from freshwater habitats change their bacterial community composition in response to the salinity pulse. The response and scope of the change in the host-associated bacterial community depend strongly on the disturbance strength since only a strong change (freshwater to salinity 6) affected the bacterial community composition. Therefore, in a host-protected environment, the strength of the disturbance determines its effect. Previous studies in other environments also identified intensity as a key feature that determines how communities respond to the disturbance (Sousa, 1984; Mccabe and Gotelli, 2000; Berga et al., 2012; Shade et al., 2012; Gibbons et al., 2016).

In this study, we also used antibiotic amended aquaria for the control manipulation since the antibiotics are known to be a very strong disturbance to bacterial communities. A clear effect on the bacterial community composition of AB-treated samples was recognized, indicating that the antibiotics treatment influenced the bacterial community strongly and differently than salinity. Antibiotics have been previously shown to change the gastrointestinal bacterial community of humans (Nogueira et al., 2019) and aquatic invertebrates (Holt et al., 2021). Our experiment shows that different stressors cause distinct reactions in the bacterial community in a host-protected environment.

Our results indicate also that the water bacterial community are disconnected from the host-protected microbiome and have little influence on its composition (Figure 7). Similar to our results, Schmidt et al. (2015) found that changes in the microbiome of a euryhaline fish are not correlated with the changes in the surrounding water bacterial communities. Therefore, deterministic processes may play a main role in composing the host-associated microbiome and leave little room for stochastic impacts (Tilman, 2004). Schmidt et al. (2015) also concluded that niche-appropriation with the best competitors at each salinity level is likely driving host-associated microbiome assembly; this mechanism might also partially explain our present findings.

#### Permanent members of the host gastrointestinal bacterial community composition

Regardless of the manipulation with different salinity levels, specific bacteria were present in all samples, suggesting an important role of these in the symbiotic relationship with the host. For example, *Mycoplasma* was one of the most abundant bacterial genera found in the snails’ gastrointestinal tract regardless of the origin (aquarium or field sites). As permanent residents of the *A. balthica* gastrointestinal tract, *Mycoplasma* may support the digestion of the algal (cellulose-based) food (Fraune and Zimmer, 2008). Genome studies of *Mycoplasma* revealed a high number of genes involved in the degradation of glycans, proteins, and complex oligosaccharides, suggesting that *Mycoplasma* supplies amino sugars and simple carbohydrates to the host (Wang et al., 2016). *Mycoplasma* has been shown to play an important ecological role for gastropods (Cicala et al., 2018) by protecting the hosts against microbial pathogen infections through sialic acid lyases that can break down the sialic acid cell-wall “coat” used by many bacterial pathogens (Severi et al., 2007; Wang et al., 2016). *Mycoplasma* has been found in various host-microbe systems including Atlantic salmon (Llewellyn et al., 2016), abalone (Cicala et al., 2018), oysters (Arfken et al., 2021), and the freshwater snail *Radix auricularia* (Hu et al., 2018).

In addition to *Mycoplasma*, *Flavobacterium*, and *Pseudomonas* were very abundant in all our snail samples. These genera are among the most commonly detected bacteria in the gastrointestinal systems of aquatic animals such as fish and aquatic invertebrates (Harris, 1993; Huber et al., 2004). Several *Flavobacteria* play a role in the mineralization of carbohydrates, amino acids, proteins, and polysaccharides in aquatic ecosystems. *Flavobacterium* as well as *Pseudomonas* are generalists and their abundant presence may reflect their broad environmental tolerance ranges and the important role of dispersal-related mechanisms in their community assembly (Yasuda and Kitao, 1980; Dempsey et al., 1989; Liu et al., 2011; Székely and Langenheder, 2014).

#### Adaptation to higher salinity of the host gastrointestinal bacterial community

The studied organism, *A. balthica*, can tolerate a salinity level of >6 in its natural environment. However, the experimental pulse salinity increase resulted in a high mortality rate. The bacterial community composition at salinity 6 in aquaria and *in situ* salinity 6 sites showed significant differences. Therefore, our results support the second hypothesis that the intestinal microbiome needs a long-term adaptation to higher salinity. Unlike the changes induced by salinity 6, the gastrointestinal microbiome responses to salinity 3 were less distinct and tended to converge on the community profiles similar to either those found in the freshwater or salinity 6. Salinity 3 is near the salinity threshold where *A. balthica* is able to regulate the osmolarity of extracellular fluids (Jordan and Deaton, 1999) and may therefore represent a critical tipping point for the host-microbiome relationship in gastropods.

Our study shows that within a host-protected environment the salinity disturbance causes the development of distinct states that depend on the strength of the disturbance. For sea level rise scenarios this indicates that an increase in salinity will also influence the host-protected microbiome if specific thresholds (in the case of gastropods, salinity ∼3) are exceeded. However, our study also showed that long-term adaptation influences the response of the host and its bacterial communities. The influence of other factors including food source, gastrointestinal physiology, and morphology, as well as the host’s gastrointestinal unique environment are expected to have a strong influence and may be subject to further studies.

## Data availability statement

The datasets presented in this study can be found in online repository: NCBI, BioProject PRJNA724976, accession OA985092–OA985113.

## Author contributions

KK and CK sampled snails, dissected the gastrointestinal tract. KK, CK, DH, and HT performed the experiments. CK did DNA extraction. CK and VK did preparation for sequencing. CK, VK, and DH did statistical and bioinformatics analysis. DH and VK did ITS sequencing and phylogenetic analysis. HT submitted sequences. IS introduced energy measurements. DH planned experiments. All authors contributed to the writing of the manuscript.
