# Supplementary material for: Distinct stages of the intestinal bacterial community of Ampullaceana balthica after salinization
Source: Front Microbiol. 2022 Aug 30;13:767334. doi: 10.3389/fmicb.2022.767334 (PMC9468257; doi:10.3389/fmicb.2022.767334)
Supplement: Supplementary file 1 [file Data_Sheet_1.docx]

Distinct stages of the bacterial community in the intestine of *Ampullaceana balthica* after the salinization

Carmen Kivistik^1^, Kairi Käiro^1^, Helen Tammert^1^, Inna M. Sokolova^2,3^, Veljo Kisand^1,4^, Daniel P. R. Herlemann^1^*

^1^Estonian University of Life Sciences, Center of Limnology, EE61101 Elva municipality, Tartu County, Estonia

^2^Department of Marine Biology, Institute for Biological Sciences, University of Rostock, Rostock, Germany

^3^Department of Maritime Systems, Interdisciplinary Faculty, University of Rostock, Rostock, Germany

^4^Institute of Technology, University of Tartu, 50411 Tartu, Estonia

* Correspondence to daniel.herlemann@emu.ee


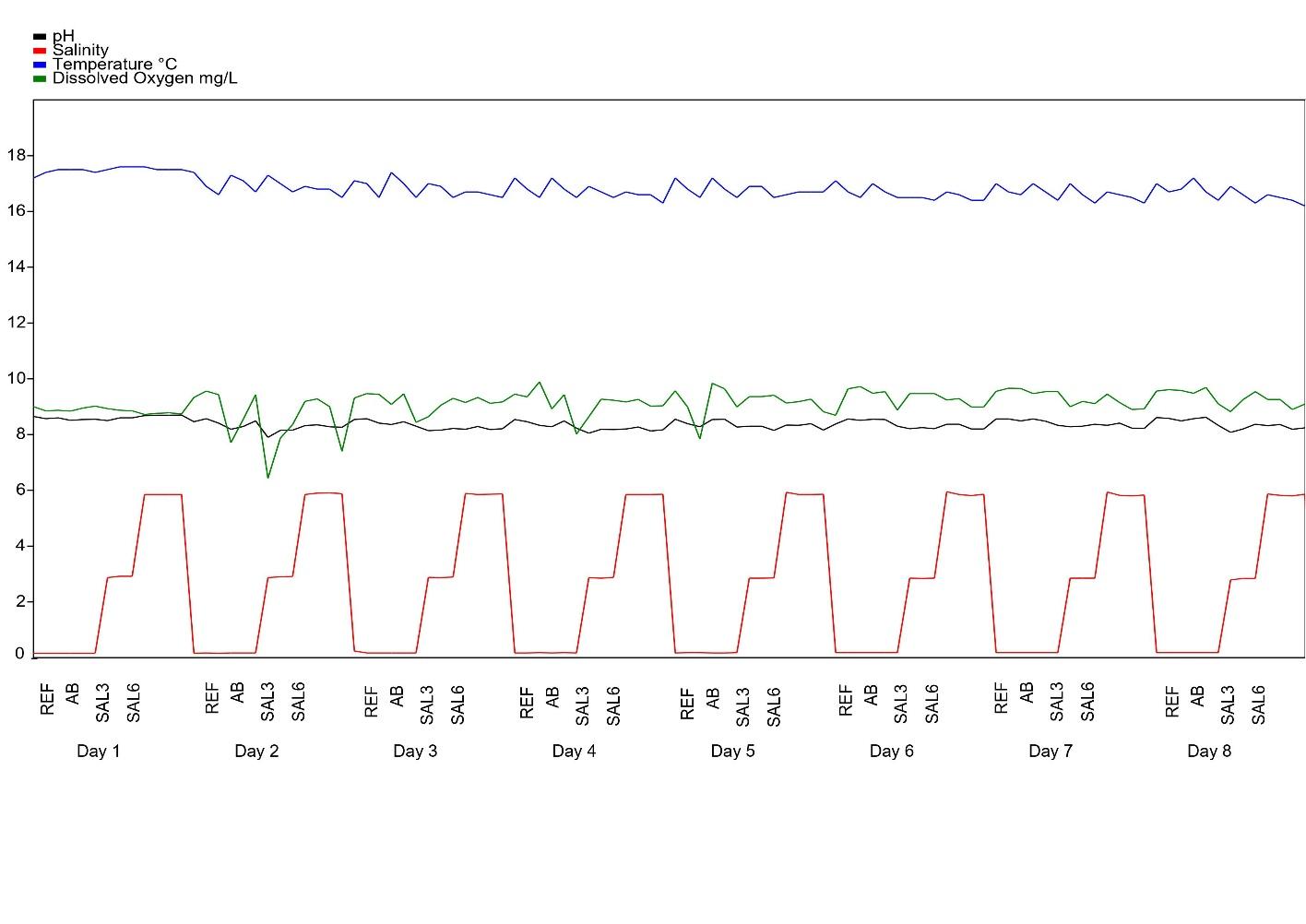


**Supplementary Fig. S1: Aquarium water condition measurements during the experiment. YSI ProDSS multisensor values for pH, salinity, temperature and dissolved oxygen from experiment day 1 til day 8.** REF – reference aquaria, AB – antibiotic amended aquaria, SAL 3 – salinity 3 aquaria, and SAL 6 – salinity 6 aquaria.


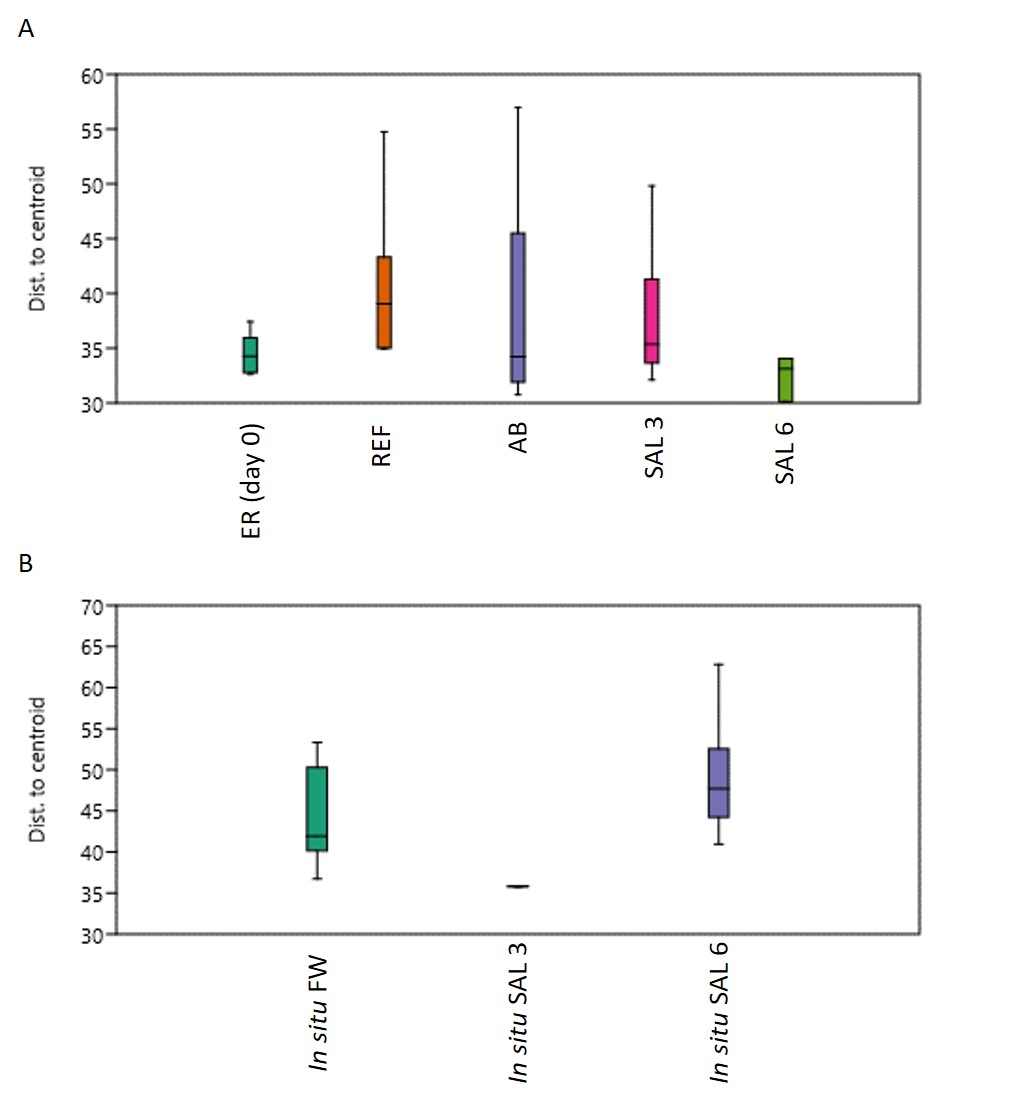


**Supplementary Fig. S2: Multivariate dispersion analysis. A: Experimental snail samples, B: *in situ* snail samples**. ER (day 0) – Snail samples from Esna River (ER) on day 0. REF – reference aquaria, AB – antibiotic amended aquaria, SAL 3 – salinity 3 aquaria, and SAL 6 – salinity 6 aquaria. *In situ* FW- samples from ambient freshwater sites (SP, SR, KL), *in situ* SAL3 – water samples from site with ambient salinity 3 (SB), *in situ* SAL6 - water samples from sites with ambient salinity 6 (RW, RE, NÕ).


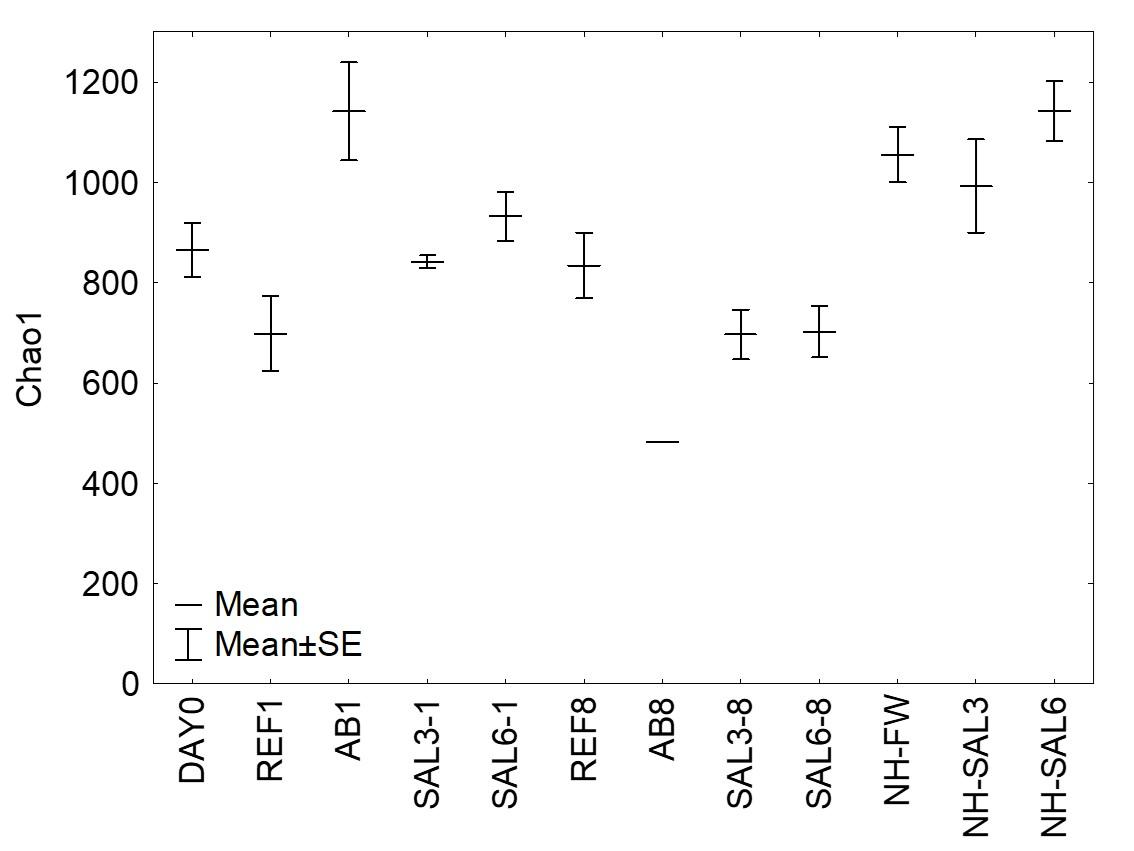


**Supplementary Fig. S3: Bacterial α-diversity of aquarium and *in situ* coastal sites water samples represented by the Chao1 index.** DAY 0 - *In situ* Esna River (ER) water samples on day 0. REF1, AB1, SAL3-1, SAL6-1 – water from aquaria on day 1; REF8; AB8; SAL3-8; SAL6-8 - water from aquaria on day 8. *In situ* FW- samples from ambient freshwater sites (SP, SR, KL), *in situ* SAL3 – water samples from site with ambient salinity 3 (SB), *in situ* SAL6 - water samples from sites with ambient salinity 6 (RW, RE, NÕ).


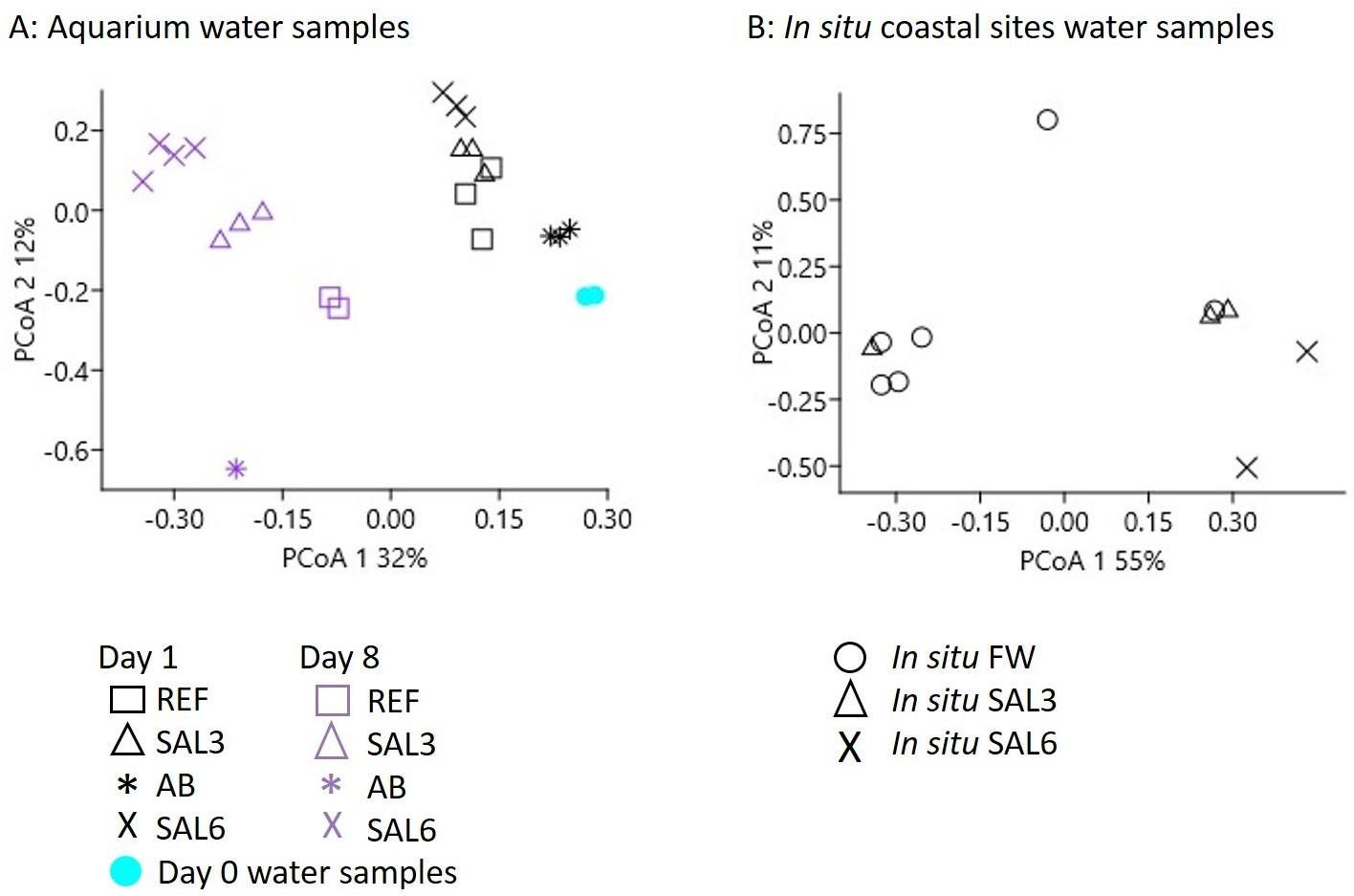


**Supplementary Fig. S4: Principal Coordinate Analysis based on Bray-Curtis dissimilarity of aquarium and *in situ* coastal sites water samples bacterial community composition on OTU level. A:** experiment aquarium water samples, **B:** *in situ* coastal sites water samples.

# Supplementary Table S1: Tukey HSD test of experiment *Ampullaceana balthica*

**energy calculations from lipid, carbohydrate and protein compounds.** The significantly different (*p*<0.01) results are marked in red.

| **Energy from lipid compounds** | | | | |
| --- | --- | --- | --- | --- |
|  | ***In situ* stream** | **REF aquarium** | **AB aquarium** | **SAL 3**  **aquarium** |
| ***In situ* stream** |  | 0.000159 | 0.000159 | 0.000159 |
| **REF aquarium** | 0.000159 |  | 0.762563 | 0.000338 |
| **AB aquarium** | 0.000159 | 0.762563 |  | 0.003666 |
| **SAL 3**  **aquarium** | 0.000159 | 0.000338 | 0.003666 |  |
| **Energy from carbohydrate compounds** | | | | |
| ***In situ* stream** |  | 0.998993 | 0.923148 | 0.999105 |
| **REF aquarium** | 0.998993 |  | 0.796801 | 0.986404 |
| **AB aquarium** | 0.923148 | 0.796801 |  | 0.932732 |
| **SAL 3**  **aquarium** | 0.999105 | 0.986404 | 0.932732 |  |
| **Energy from protein compounds** | | | | |
| ***In situ* stream** |  | 0.000160 | 0.000160 | 0.000160 |
| **REF aquarium** | 0.000160 |  | 1.000000 | 0.001291 |
| **AB aquarium** | 0.000160 | 1.000000 |  | 0.001008 |
| **SAL 3**  **aquarium** | 0.000160 | 0.001291 | 0.001008 |  |
